# VirIoT: A Cloud of Things That Offers IoT Infrastructures as a Service

**DOI:** 10.3390/s21196546

**Published:** 2021-09-30

**Authors:** Andrea Detti, Hidenori Nakazato, Juan Antonio Martínez Navarro, Giuseppe Tropea, Ludovico Funari, Luca Petrucci, Juan Andrés Sánchez Segado, Kenji Kanai

**Affiliations:** 1 CNIT—Electronic Engineering Department, University of Rome “Tor Vergata”, 00133 Rome, Italy; giuseppe.tropea@cnit.it (G.T.); ludovico.funari@uniroma2.it (L.F.); luca.petrucci@uniroma2.it (L.P.); 2 Department of Communications and Computer Engineering, Waseda University, Tokyo 169-0072, Japan; nakazato@waseda.jp (H.N.); k.kanai@aoni.waseda.jp (K.K.); 3 Odin Solutions, Alcantarilla, 30820 Murcia, Spain; jamartinez@odins.es (J.A.M.N.); jasanchez@odins.es (J.A.S.S.)

**Keywords:** IoT, cloud computing, test-bed, interoperability

## Abstract

Many cloud providers offer IoT services that simplify the collection and processing of IoT information. However, the IoT infrastructure composed of sensors and actuators that produces this information remains outside the cloud; therefore, application developers must install, connect and manage the cloud. This requirement can be a market barrier, especially for small/medium software companies that cannot afford the infrastructural costs associated with it and would only prefer to focus on IoT application developments. Motivated by the wish to eliminate this barrier, this paper proposes a *Cloud of Things* platform, called VirIoT, which fully brings the Infrastructure as a service model typical of cloud computing to the world of Internet of Things. VirIoT provides users with virtual IoT infrastructures (Virtual Silos) composed of virtual things, with which users can interact through dedicated and standardized broker servers in which the technology can be chosen among those offered by the platform, such as oneM2M, NGSI and NGSI-LD. VirIoT allows developers to focus their efforts exclusively on IoT applications without worrying about infrastructure management and allows cloud providers to expand their IoT services portfolio. VirIoT uses external things and cloud/edge computing resources to deliver the IoT virtualization services. Its open-source architecture is microservice-based and runs on top of a distributed Kubernetes platform with nodes in central and edge data centers. The architecture is scalable, efficient and able to support the continuous integration of heterogeneous things and IoT standards, taking care of interoperability issues. Using a VirIoT deployment spanning data centers in Europe and Japan, we conducted a performance evaluation with a two-fold objective: showing the efficiency and scalability of the architecture; and leveraging VirIoT’s ability to integrate different IoT standards in order to make a fair comparison of some open-source IoT Broker implementations, namely Mobius for oneM2M, Orion for NGSIv2, Orion-LD and Scorpio for NGSI-LD.

## 1. Introduction

Nowadays ubiquitous, cloud computing decouples infrastructure providers from application developers by offering the “computing” infrastructure as a service. The huge proliferation of web, mobile and machine-to-machine applications is undeniably an effect of this decoupling. It allows software companies to focus their efforts solely on the development of applications without needing to worry about managing the supporting computing infrastructure.

This paper proposes a *Cloud of Things* platform, named VirIoT (from “Virtual IoT”), that brings the infrastructure as a service model to the world of the Internet of Things, precisely matching an equivalent goal. The platform offers sensor/actuator virtualisation, as well as interoperability and support to current IoT standards, to create the concept of virtual IoT infrastructures in the cloud that improves the current state of the art [1,2]. Indeed, although cloud computing providers (e.g., Amazon AWS [3] or Microsoft Azure [4]) offer IoT services, they only help with the collection, distribution and processing of IoT data, but the IoT infrastructure that produces and consumes that data is currently outside the cloud and must usually be deployed, connected and managed by application developers themselves [5,6]. The need to own infrastructure can be a market barrier, especially for small/medium software companies that cannot afford the related costs. For these companies, it might be beneficial to focus only on IoT application development and possibly rent the necessary IoT sensors and actuators. Accordingly, the proposed Cloud of Things platform can be used by a cloud provider to extend its portfolio of IoT services, offering developers the possibility of renting tailored IoT *virtual* infrastructures.

In order to introduce our work, in what follows we first provide a background on how IoT applications these days are tightly coupled to their supporting infrastructure. Then, we discuss how our Cloud of Things evolves this scenario by decoupling application developers from infrastructure providers.

### 1.1. Background

**Figure 1:**
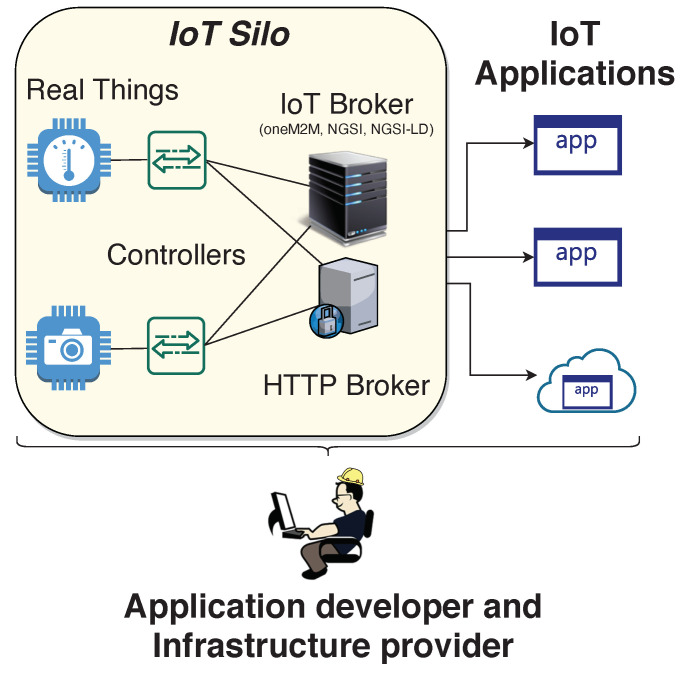
IoT applications based on IoT Silos.

Figure 1 shows a typical scenario used nowadays to run IoT applications [7]. There is an IoT infrastructure, also known as *IoT Silo*, composed of *(Real) Things*, *Controllers* and *Brokers*. Controllers are software components that act as adapters of interfaces and data gateway between the things and upstream endpoints that are either the destination *IoT Applications* or intermediate brokers providing data integration services and often supporting more advanced functionality that is transversal to IoT Applications, such as data aggregation and querying, storage and publish/subscribe/notify and access-control. Applications, which live outside of the Silo, can be implemented from scratch or may leverage IoT “computing” services offered by cloud providers. In this scenario, the application developer must usually manage not only the application but the IoT Silo infrastructure as well, thus playing the additional role of infrastructure provider.

In Figure 1, we consider two kinds of brokers, HTTP and IoT, which manage access to two classes of IoT information: *generic contents* and *context data*, respectively.

Generic contents are heterogeneous (large) data pieces, such as images or video streams, produced by the Silo’s things and exposed as HTTP resources. Applications access these contents either directly or through the mediation of an *HTTP Broker* (or proxy) that provides access-control and other features, such as data caching and API gateway functions.

Context data pieces are a few bytes long and digitally represent an entity with its properties, such as the status of a lamp or the temperature of a room. Silo’s things manage such context data, and applications access it either directly or through a server, usually called *IoT Broker*. The IoT Broker is a kind of integration platform that manages the life-cycle of context data and offers generic back-end services such as the following: data storage, updates, queries, subscriptions, access control, etc. For the services to be successfully delivered, context data must be homogeneous, i.e., compliant with a specific information model. When a Silo’s thing does not natively conform to the IoT broker information model and API, a Controller (or agent) is required to provide the necessary adaptation.

Many IoT standardization efforts precisely focus on defining the information model and API of their IoT Brokers, and some representative standards are oneM2M [8] and FIWARE NGSIv2 [9], which are evolving towards ETSI NGSI-LD [10] where there are better supported linked data and semantics.

The information models of these standards are based on generic concepts that allow representing the context data of a wide range of use cases. For example, the NGSI-LD information model’s main concepts are Entities, with Properties and Relationships with other Entities (we use the upper case notation for Entity, Property and Relationship when referring specifically to NGSI-LD concepts). It is based on the JSON-LD syntax and its idea of Linked Data. Listing 1 shows a NGSI-LD Entity that digitally represents a “person counter”. Its Properties are as follows: daily persons, for which its value is the number of people that have passed through the counter; and location, which contains a description of the gate where the counter is located. The Entity also has a unique id, a type and a @context semantic annotation typical of JSON-LD.

**Listing 1:**
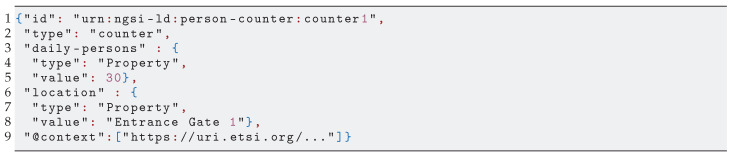
NGSI-LD Entity representing a person counter’s data.

### 1.2. Proposed Evolution

Overall, commercial solutions and many studies in literature (see [11,12,13,14,15] for instance) involving cloud and IoT focus primarily on computing functionality, such as the following: efficient virtualization of computing and network resources, analytics, flow programming, data collection and so on. Our Cloud of Things platform, VirIoT, is *complementary* to this state of the art because it focuses on virtualizing IoT infrastructures (Silos) that contain things that can in turn be connected to the aforementioned computing capabilities, such as the IoT services offered by Microsoft Azure or Amazon Web Services.

**Figure 2:**
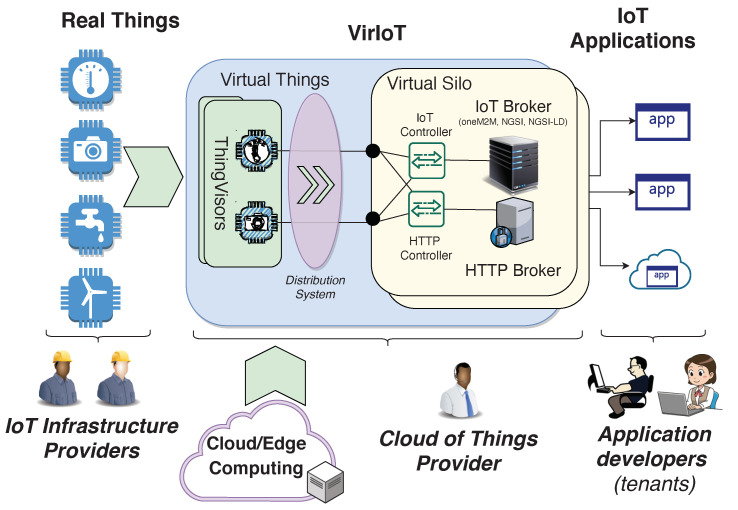
IoT Applications based on VirIoT services.

Figure 2 shows how VirIoT evolves the scenario in Figure 1. The application developer is no longer alone, but a Cloud of Things provider and IoT infrastructure provider also join the scene. The Cloud of Things provider runs VirIoT on top of a cloud/edge computing platform to create *Virtual Things* (vThings) and *Virtual Silos* (vSilos). A vThing is an emulation of a real thing, and the emulation process is carried out by a software component named *ThingVisor* that interacts with external things offered by IoT Infrastructure providers. A ThingVisor can implement one or more vThings, and many ThingVisors can be executed in the platform. vSilos are isolated IoT infrastructures created at the request of application developers (tenants) and to which they can connect the vThings needed for their applications in a “drag-and-drop” fashion. The services of the connected vThings, such as the provision of sensor data or the execution of actions, can be accessed by the applications through the mediation of dedicated IoT and HTTP Broker servers running in the vSilos. A tenant has freedom in choosing the technology of her vSilo’s IoT Broker, among a portfolio of possible ones (e.g., Brokers implementing the oneM2M standard, Brokers for NGSIv2, etc.). Therefore, VirIoT is able to integrate different IoT standards into a single virtualization platform. A vSilo also contains an HTTP Controller and an IoT Controller, which takes care of the specific data+control plane functionality for their related Brokers.

Thus, in a nutshell, VirIoT completely decouples IoT infrastructure providers from application developers through a full virtualization of Things and Silos in the cloud (or edge).

**Figure 3:**
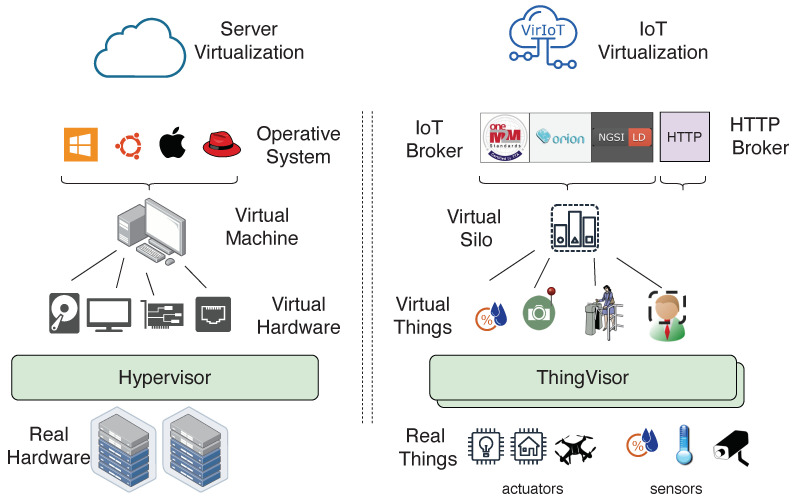
Server Virtualization vs. IoT Virtualization.

Figure 3 shows a parallelism between the concepts of server virtualization, specific to Cloud Computing, and IoT virtualization, specific to VirIoT. Server virtualization uses a pool of real hardware to offer virtual machines made of a configurable set of virtual hardware (virtual memory, disks, CPUs, etc.) that applications can access through a guest operating system that each tenant can chose. The virtualization process is made by an Hypervisor, a software that interacts with real hardware to create the virtual one. Similarly, the VirIoT virtualization stack uses a pool of real things to offer vSilos made of a configurable set of vThings that can be accessed through two Brokers working in synergy for which its IoT technology can be chosen by each tenant. The thing virtualization process is performed by a ThingVisor.

Making an explicit comparison, real things are akin to real hardware, ThingVisor to Hypervisor, vThings to virtual hardware, the Brokers to the operating system and the vSilo to the virtual machine.

A use case of VirIoT involves the development of large-scale IoT applications, such as those in smart cities, that require broad infrastructure (for example, those consisting of thousands of cameras or sensors). Software companies that cannot afford high installation costs can simply rent a virtualized (possibly scaled-down and much more easily manageable) version of these things from a VirIoT-enabled Cloud of Things provider. At the same time, the large companies or agencies that have deployed and own the IoT infrastructure can partially recoup their upfront costs by selling access to their things to the VirIoT-enabled Cloud of Things provider, which uses them to create many Virtual Things to sell to multiple tenants.

Another use case involves running VirIoT in your own IoT infrastructure, similar to a private cloud where the infrastructure provider, cloud provider and application developer are one and the same. This model allows different applications to use different vSilos, thus being completely isolated from each other because they do not share any Brokers, and this increases the reliability of the system because no application, malicious or benign, can affect data in a Broker that is used by other applications. In addition, the developer of an application can choose the IoT Broker that best suits its needs: for example, a faster IoT Broker with a limited API if the application needs low latency; or a slower IoT Broker but with a powerful API if it needs complex queries based on the semantic aspect of the data, for instance.

All in all, the novelty of the proposed IoT virtualization is two fold: first, the introduction of a new virtualization model for IoT applications, which fully exploits the cloud, efficiently multiplexes resources and minimally impacts the consolidated development workflow. It simplifies their deployment and expands the service portfolio of cloud providers. Second, the design and implementation of an open-sourced cloud-native architecture; it leverages existing technologies to deliver the above virtualization model at scale through micro-services that virtualize all components of the typical IoT application. In the following sections, we review related works, we present the VirIoT architecture and we report a performance evaluation of a wide area VirIoT instance that involves data centers in Europe and Japan. Finally, we draw conclusions.

## 2. Related Works

Regarding infrastructure-based technologies, investigations such as the one from Usman et al. [2] propose a pure infrastructure-based approach where they use a Data Center based on OpenStack. In this manner, thanks to the deployment of a specific OpenStack component in the IoT device, as well as with using what they call a Sensor Data Transfer Service, the information coming from an IoT device can be introduced into the OpenStack Controller Node by using an IoT Data Acquisition Server, which stores the data coming from the devices into an centralized InfluxDB. Stack4Things, by Longo et al. [16], also goes in this direction regarding infrastructure management. This proposal adopts OpenStack as well, introducing a novel component for provisioning configurations, tasks for board-hosted sensing and actuation resources. Finally, as mentioned in Section 1, the industry has also moved to the Cloud IoT paradigm because of the high potential it brings regarding both heterogeneity, the high quantity of information which can be processed and the business potential. Examples are Google Cloud IoT, Amazon Web Services for IoT and Microsoft Azure. They have been compared in terms of features and performance by Pierleoni et al. [17]. Nevertheless, they are more focused on how IoT information coming from devices is integrated or processed and do not consider cross-domain interoperability or virtualisation of Things within the infrastructure as a primary goal.

Concepts such as IoT virtualization or cloud of sensors have already been discussed in literature. One of the first works was by Kabadayi et al. [18] in 2006, where they envisioned the idea of virtualizing sensors with the objective of decoupling the acquisition of the information from the use of this information by end users. Within this scope, we also find works from Alam et al. [19] where they proposed a layered architecture where the information obtained from IoT devices is enriched by using semantic representations. Nevertheless, since this work does not use any established data-modeling international standards for representing the information, a specific set of concepts was defined. Thus, it was possible to represent a limited set of type of things only, making this work not easily scalable or extensible concerning this aspect.

In 2014, Aazam et al. [20] addressed the importance of the IoT and the combination of IoT information with cloud computing. In the architecture they propose, they not only provide remarks on the use of a middleware layer with the purpose of storing data but also of performing some processing tasks over the information gathered from the IoT level. This aspect is also considered by Madria et al. [21] where they also introduced the possibility of having different configurations, such as one-to-many, many-to-one or many-to-many, for the use of a virtual sensor representation where an intermediate processing task could be performed too.

Other works in IoT virtualization were made by Dar et al. [22], for instance, where the main focus was the inherent properties of IoT devices regarding link instability, availability and the likes so that a platform could provide this information to the users through the creation of different services, which receive users requests, and these services are the ones receiving information coming from the sensors.

More recently, in 2019, Alam et al. [23] provided a survey for software definition and function virtualization. In this paper, the authors address the issue of IoT virtualization from the point of view of technologies such as Software Defined Networks and Network Function Virtualization. Basically, they provide an architecture where these concepts are integrated so that IoT data can be processed dynamically.

Unlike the works we reference above, our position is novel in that it extends the scope of virtualization from the data acquisition (in the ThingVisor) up to the vSilo, employing also a standard representation of information based on NGSI-LD. VirIoT includes in a single virtualization instance (the vSilo) not only sensors but also a data integration platform whose technology can be a user’s choice. Therefore, the resulting solution embraces many IoT standards into a single platform and consequently addresses the inevitable interoperability issues. Moreover, VirIoT offers *Things as a Service*, i.e., not only virtual sensors but also virtual actuators while handling an efficient distribution of context data and generic heterogeneous contents.

Other aspects we would like to highlight concern the different networking and computing technologies, which can be found in the literature, that are also inspired the design of our VirIoT platform, especially for vSilos, so that virtual things could be offered to end-users, tailoring the information according to their specific needs.

The NIST [24] has defined the term *cloud computing* in 2011 as a model for provisioning and releasing a shared pool of computing resources in a quick manner with minimal effort or service provider interaction. This definition also talks about some essential characteristics such as on-demand self-service, broad network access or rapid elasticity.

In addition, more recent works such as Petrakis et al. [11], developed an “as a Service” framework, based on micro-services, for the IoT. Unlike other proposed solutions they also consider a framework covering edge and cloud services, where an IoT gateway may collect information and send it to a cloud back end to support data sharing, storage and processing.

Samaniego et al. [15] also relies on the same idea of taking advantage of edge nodes and their performance capabilities so that IoT services could be more efficiently provided. For this purpose, they proposed an architecture for virtual systems comprising sensor, fog and cloud layers that collaborate to provide IoT services at the edge.

Li et al. [12] exploits object virtualization by using network function virtualization as a flexible mean for network service provisioning. The authors propose a layered framework encompassing smart objects, fog and cloud to overcome the obstacles resulting from resource constraints on sensory-level nodes. This work considers the idea of exploiting not only cloud services but also having a flexible approach to distribute processing tasks to other planes/levels such as edge or fog. In this manner, the resources dedicated to the processing tasks can be virtualized and dynamically deployed, optimizing them for better performance, thus making for a scalable approach. These ideas are also used in the solution we propose thanks to the dynamic deployment and orchestration capabilities of Kubernetes, but we also devised a data distribution system that is able to follow the network topology of the dynamic deployment of ThingVisors and vSilos.

Table 1 summarizes the related-works analysis we carried out.

## 3. VirIoT Architecture and Services

VirIoT is an open-source microservices architecture [25] whose *services* (ThingVisors, vThings, vSilos, Master Controller, etc.) are implemented as Docker containers. Their deployment is managed by an underlying Kubernetes (k8s) cluster [26], possibly spreading over a wide geographical area of central and edge data centers. VirIoT offers edge-computing functionality, meaning that it is possible to control *where* ThingVisors and vSilos can be deployed. In this manner, tenants can deploy their vSilos at a specific data center closer to the final applications, with obvious advantage in terms of latency and bandwidth consumption. For the same reason, the administrator can deploy ThingVisors near the real things that interact with them.

**Figure 4:**
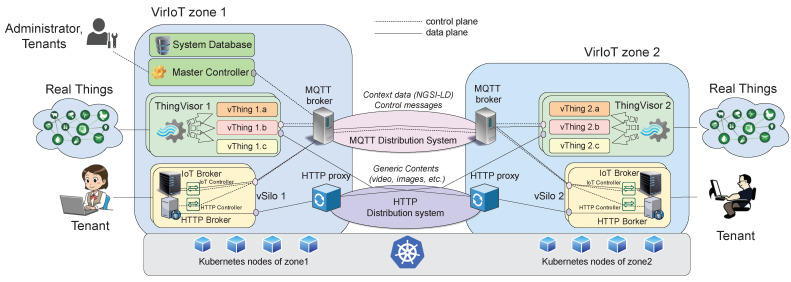
VirIoT system architecture.

Figure 4 shows the architecture of VirIoT and its relationship with an underlying k8s cluster used for the dynamic deployment of VirIoT services. The k8s cluster is organized in *zones* where VirIoT services run. A zone can be a central or edge data center for which its nodes have specific k8s labels (viriot-zone) that allow discriminating them and controlling which zone to install each service. An MQTT-based [27] distribution system and an HTTP-based distribution system, consisting of MQTT brokers and HTTP proxies, are used to efficiently transport context data and generic contents among ThingVisors/vThings and vSilos with low latency. In addition to this “data plane” information, VirIoT services also exchange control information by using the MQTT distribution system. The main service of the “control plane” is the *Master Controller*, which exposes a REST API to control the life cycle of ThingVisors and vSilos, and it stores the system status in a NoSQL *System Database* that is MongoDB.

The platform installation requires labeling the involved k8s nodes with specific labels and the manual deployment of Master Controller, System Database and MQTT/HTTP Distribution Systems through specific k8s YAML files. After that, resource discovery and platform configuration operations can be performed either through the REST API exposed by the Master Controller or a command-line interface tool (CLI) (see [25,28]). For example, the list of vSilos types (Docker images named *Flavour*) that a tenant can instantiate, and the list of available vThings can be displayed; moreover, ThingVisors can be added and configured, and vSilos can be instantiated and configured.

Regarding the security aspects, we currently considered as trusted the infrastructure within a data center, so we did not apply any encryption for internal data transfer. Data encryption is used on VPN links connecting cloud/edge sites to each other. Regarding external communications between ThingVisors and real things and between vSilos and applications, we leverage the security mechanisms (e.g., HTTPs with digital certificate) offered by real things and vSilos IoT Brokers, respectively, as we do not want to change them but integrate them as they are. In terms of platform configuration control, the Master Controller includes “basic” access control functionality based on JSON Web Tokens, which classifies clients as administrators and tenants. Tenants can control and see only their vSilos, while the administrator can enrich the portfolio of vSilos and ThingVisors that the platform can offer. Finer access control schemes can be placed on top of the basic one as discussed in [29].

The following subsections describe some key concepts about the internal information model and interoperability, and then we proceed deeper into the main services of the architecture: ThingVisors, vSilos and Distribution Systems.

### 3.1. Information Models and Interoperability

Within VirIoT, we used the NGSI-LD information model [10]. A vThing exposes its context data by NGSI-LD Entities with Properties whose values change over time. Entities are encapsulated and continuously transferred from a vThing to all vSilos connected to it so that they have a copy of the latest version of the Entities.

**Figure 5:**
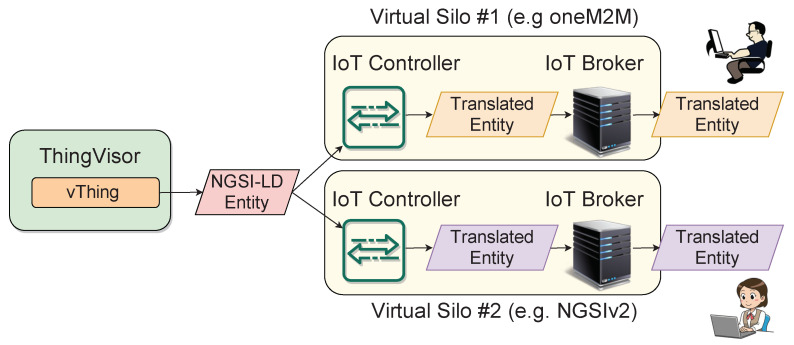
Interoperability.

A tenant accesses vThing’s context data through the vSilo’s IoT Broker, which may adopt different technologies from NGSI-LD. Therefore, there is an interoperability issue, i.e., a need for a NGSI-LD to X “translation”, where X is the information model used by the destination IoT Broker. As shown in Figure 5, this translation between information models is handled within the vSilos. The context data are translated from NGSI-LD into the IoT Broker’s information model upon arrival by the IoT Controller. We depict a scenario where two vSilos use two different Brokers: a oneM2M IoT Broker and an NGSIv2 IoT Broker. Tenants have connected the same vThing to their vSilos, for instance, a virtual person counter. The person counter’s context data are represented by the NGSI-LD Entity in Listing 1. The data reaches the vSilos, and it is thereby translated to oneM2M (Listing 2) and NGSIv2 (Listing 3), respectively, by the IoT Controllers.

Thus, we addressed this issue of interoperability between IoT information models through the mediation of NGSI-LD as a kind of *neutral-format* that makes it easy to translate to other information models with negligible loss of information. This approach allows decoupling between ThingVisors’ and vSilos’ developers, who only need to take care of translation to/from NGSI-LD; this simplifies the continuous integration and evolution of the platform towards new types of vSilos, ThingVisors and vThings.

**Listing 2:**
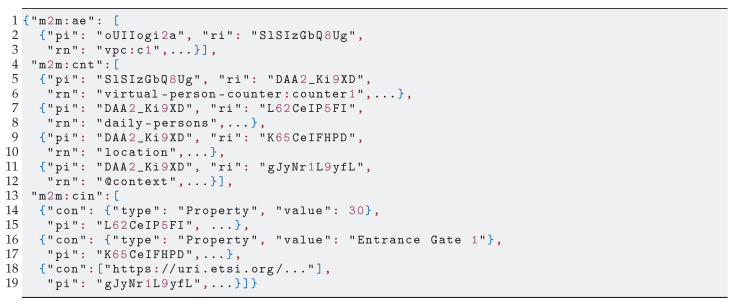
Context data. from Listing 1 translated to oneM2M.

**Listing 3:**
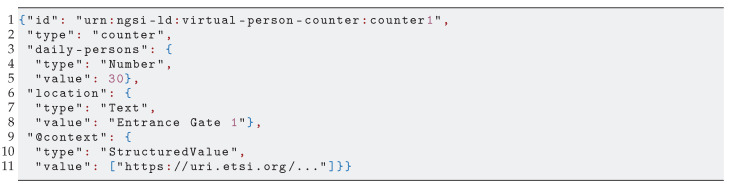
Context data from Listing 1 translated to NGSIv2.

Concerning the choice of NGSI-LD as neutral-format, our experience from several real-life IoT applications and data (models), plus the current state of IoT standardization initiatives pushing NGSI-LD as a layer on top of existing standards [30], leads us to that choice. Moreover, we have been able to develop mapping rules, shown in Table 2, between NGSI-LD and two widespread IoT standards: oneM2M and NGSIv2. We used these rules in the IoT Controllers we implemented. Listing 2 (some keys removed for clarity) and Listing 3 show how NGSI-LD-based context data of a vThing are translated to oneM2M and an NGSIv2 by the respective set of rules.

### 3.2. ThingVisors, vThings and Thing Virtualization Concept

Thing virtualization is a core concept of VirIoT, and our position is that the virtualization of a thing is about the following:

***Creating the illusion that a thing is present by producing the data and provoking the actions it would have produced or provoked if it was real.***

**Figure 6:**
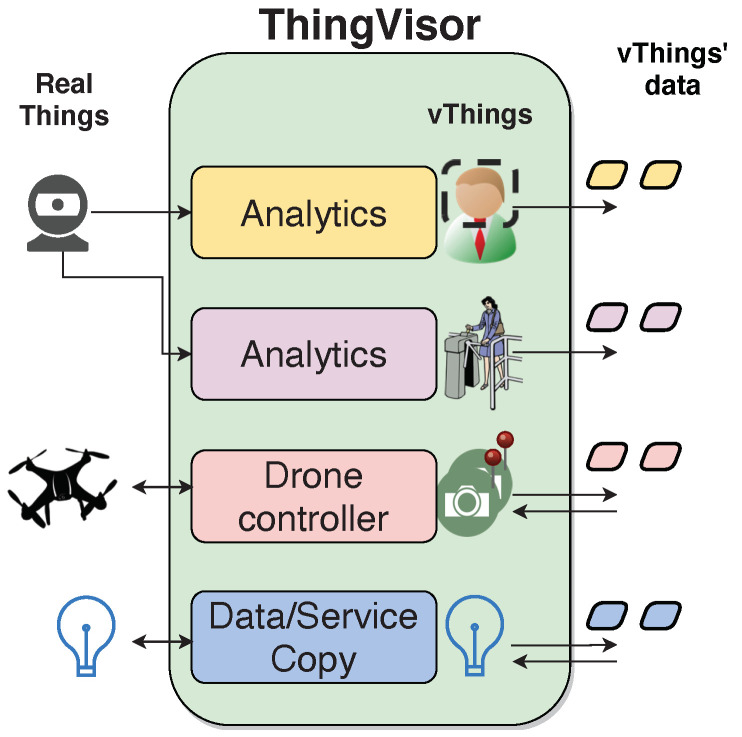
ThingVisor and vThings.

Figure 6 illustrates an example of our concept of thing virtualization. We observe a single ThingVisor that implements a set of vThings by interacting with real things living in the external environment. A real thing can be a sensor (as a camera) that sends data to the ThingVisor, and the data are used to generate data of one or more vThings. A real thing can also be an actuator (such as a drone or a lamp) that is controlled by the ThingVisor in order to create the data or perform the actions of one or more vThings.

In Figure 6, the vThings are a *face detector* that a tenant can configure by passing to it the parameters of a target face to be detected, a *person counter* that monitors the number of people passing through a gate, a *pool of cameras* placed in specific locations of interest that take pictures at a slow rate (e.g., one every 15 min) and a *lamp* for which its on/off status can be actuated. Thing virtualization is made by different functionalities (analytics, drone controller, etc.) running in the ThingVisor. Starting with the virtualization of the lamp, this is an example of “transparent” virtualization whereby the services and data of a real thing are one-to-one twinned with those of a vThing. In this manner, any thing that can be connected to the VirIoT platform can be virtualized and used by tenants. The person counter and face detector virtualizations are achieved by processing images coming from the same camera. Thus, a single real thing can be used for many vThings. Finally, the virtualization of the pool of cameras is achieved by controlling the path of a drone to periodically take a picture over the locations where virtual cameras would be placed if they were real. Therefore, these examples have shown that the thing virtualisation concept that we are considering in the scope of this paper may go beyond a fetch-and-process data modus-operandi [21] since it can also involve the control of real things, such as drones or more generic actuators.

Now that we have explained our concept of Virtual Thing and illustrated how virtualization can be performed, we describe the interactions among vThings and vSilos. We differentiate two main classes of vThings: Virtual Sensor and Virtual Actuator. A Virtual Sensor is a vThing that only has sensing capabilities. A Virtual Actuator is a vThing that has actuation capabilities too. Indeed, many actuators also have properties that can be monitored, such as the current on/off status of a lamp. For this reason, we consider that actuators may also include sensing functions.

#### 3.2.1. Virtual Sensors

**Figure 7:**
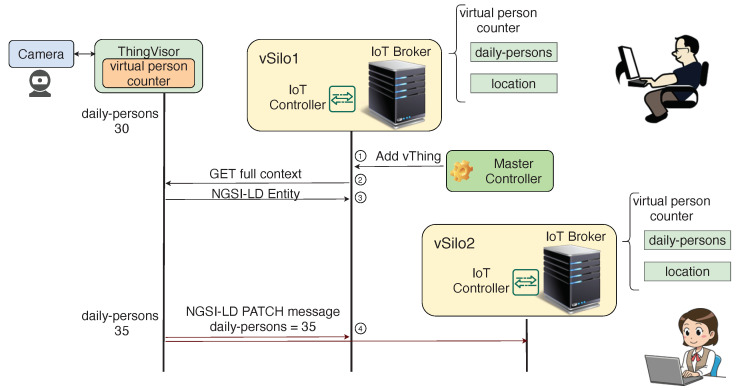
Virtual Sensor workflow.

A Virtual Sensor produces context data and, possibly, also generic contents such as JPEG images. The vSilos *pull* generic contents via HTTP as soon as tenants request them. For context data, Virtual Sensors promptly *push* context data to vSilos by using an incremental approach, as shown in Figure 7, where there are two vSilos that use the “person counter” Virtual Sensor. Initially, only vSilo2 is connected to the Virtual Sensor. Then, using the Master Controller, vSilo1’s tenant adds (connects) the same Virtual Sensor to his vSilo too (step 1). As a result, the complete set of context data shown in Listing 1 is fetched by vSilo1 as a consequence of the GET full context request control message sent to the vThing by the IoT Controller (steps 2 and 3); subsequently, when an internal Property of the Virtual Sensor changes, e.g., the value of daily-persons increases from 30 to 35, the Virtual Sensor sends a kind of context update message to the connected vSilos, called NGSI-LD PATCH message (step 4). As shown in Listing 4, the payload of this message contains the modified Properties only.

**Listing 4:**
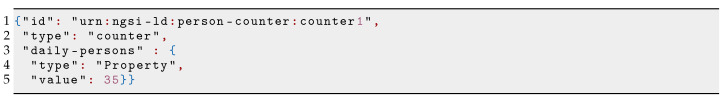
NGSI-LD PATCH message updating the daily-person Property of the Entity in Listing 1 from 30 to 35.

#### 3.2.2. Virtual Actuators

In addition to producing generic contents and context data representing its properties, a Virtual Actuator also performs actions whenever it receives *actuation-commands*. Let us explain how tenants trigger actuation by using a generic example involving a simple “virtual lamp” Virtual Actuator.

Listing 5 shows the context data associated with a virtual lamp, which includes two Properties: on, whose value is the status of the lamp (false means lamp off); and commands, whose value is a list of possible actuation-commands. In this case, this list is composed of set-on and token-req commands that can be used to configure the on status of the lamp and to request an authorization token, respectively.

**Listing 5:**
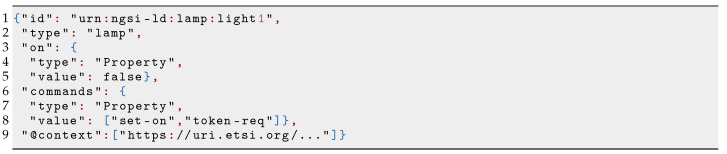
NGSI-LD of a lamp.

**Figure 8:**
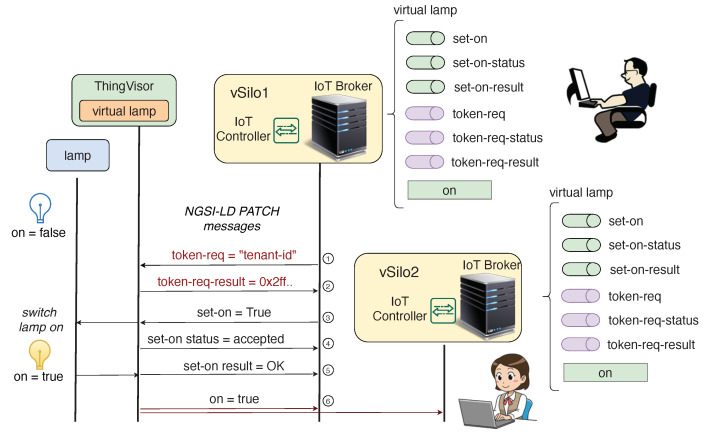
Virtual Actuator workflow; QoS = 2.

Figure 8 shows the interaction between tenants and the virtual lamp, resulting in the twinned real lamp changing its state from off to on. We have two tenants who have the virtual lamp in their vSilos whose its initial state is off (on=false). For each actuation-command, a vSilo exposes its IoT Broker three *actuation-pipes* used by the tenant to inject the actuation-command and receive feedback messages (the implementation of an actuation-pipe depends on the IoT Broker, for example: in oneM2M, an actuation-pipe is implemented by a oneM2M container; and in NGSIv2/NGSI-LD, an actuation pipe is implemented by an Entity. The IoT Controller is made aware of the actuation-pipes to be created via the commands Property).

To switch on the lamp, the tenant of vSilo1 obtains an authorization token to execute the command as discussed later on (steps 1 and 2) and then injects the actuation-command into the set-on actuation pipe, shown in Listing 6. The command is immediately received by the IoT Controller and then transferred to the ThingVisor that implements the virtual lamp (step 3). When the virtual lamp receives the message, the actuation-command is accepted and the virtual lamp starts the actuation process of the real lamp by using the proprietary API the lamp provides.

When the actuation-command is accepted, a *status* feedback is sent to vSilo1 only to inform the tenant that the actuation is in progress (step 4). The feedback message is received by the IoT Controller and relayed to the set-on-status actuation-pipe of the IoT Broker from which it can be observed by the tenant.

At the end of the actuation, when the lamp is switched on, a *result* feedback is sent to vSilo1 only to inform the tenant that the actuation is complete (step 5). This feedback message is relayed from the IoT Controller to the set-on-result actuation-pipe of the IoT Broker. Moreover, since the context Property on is changed, as a result of the actuation from False to True, a context update message is sent to all vSilos, as previously explained (step 6).

**Listing 6:**
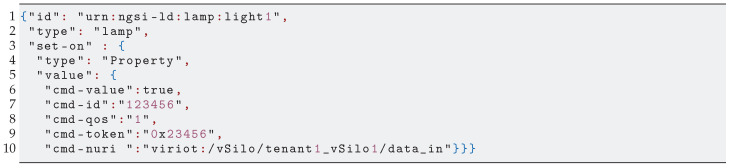
set-on actuation-command.

Actuation commands and status/result feedback are simple messages, and they are encoded by NGSI-LD PATCH messages. For example, the message in Listing 6 represents the set-on actuation-command as a NGSI-LD Property whose value is a JSON object containing the following attributes:

- cmd-value contains the arguments of the command;
- cmd-id is a unique ID of the command;
- cmd-qos is a concept of “actuation QoS” we introduced to differentiate the types of feedback messages sent by a Virtual Actuator: 0 = no feedback; 1 = result message at the end of the actuation; 2 = one or more status messages during the actuation and a result message at the end of the actuation. The value QoS = 0 is useful for use cases where actuation commands are issued at such a high rate that waiting for feedback is useless. The value QoS = 1 is useful for fast and reliable actuation. The value QoS = 2 is useful for a long lasting actuation that requires feedback during execution. For example, for the virtual face detector, an actuation-command set-face-feature is used to send the parameters of the face to be detected, QoS is set to 2 and status messages are sent to the requesting vSilo each time the face is detected. We used QoS = 2 for the lamp example for completeness, but QoS = 1 is more appropriate for this use case;
- cmd-token is an *authorization token* optionally used to manage conflicting commands, as discussed below;
- cmd-nuri is a notification URI (nuri) where to send the actuation feedback, and it is the data_in topic of the vSilo by default (see Section 3.4.1) so that only the requesting vSilo will receive feedback messages.

Status/result feedback messages are identical to the actuation-command they refer to, but they have additional cmd-status/cmd-result keys.

We note that Virtual Actuators can receive conflicting commands from different tenants. The access-control policy that handles conflicts obviously depends on the type of actuator. For example, if a group of tenants rent a room where there is a lamp exposed as a vThing, the control of this virtual lamp is granted equally to all tenants in the group. Thus, the ThingVisor of the virtual lamp implements a group-oriented access-control policy. Differently, other Virtual Actuators may need priority-oriented access-control policies for which many tenants can send actuation-commands, but those commands may or may not be accepted by the ThingVisor depending on the tenant’s priority and the current state of the Virtual Actuator. For example, if a tenant has been granted permission to control a virtual drone, any other tenant with lower or equal priority cannot receive this permission, while any other tenant with higher priority can override the previously granted permission and start controlling the drone. These examples show that different Virtual Actuators may need different access-control policies. Consequently, we preferred to include in VirIoT only the means to carry access-control policy decisions (i.e., the cmd-token) but left the implementation of the necessary policy within the specific ThingVisor and, thus, in the hands of the Virtual Actuator developer. This makes VirIoT flexible in terms of including actuators with heterogeneous (and unforeseen) policies.

The procedure for obtaining an authorization to execute an actuation-command, or more broadly to control a Virtual Actuator, is (again) a ThingVisor implementation choice, since the information needed to obtain the authorization depends on the specific access-control policy. Figure 8 shows a possible example of this procedure that follows a token-based access control scheme [31]. The virtual lamp exposes a specific actuation-command token-req, which is used by a tenant to request a token to be used in any subsequent actuation-command (e.g., set-on) in the cmd-token field (step 1). Information about the tenant, for example, the tenant ID, is included in the cmd-value field of token-req. When the ThingVisor receives the token-req, the access-control policy is used, and eventually a token is sent back with the token-req-result message (step 2). To some extent, VirIoT reuses the same approach as HTTP in which the authorization header is the means of carrying the result of an access-control policy actually implemented in the web server and outside the scope of the HTTP protocol.

As a proof-of-concept of the proposed actuation-model, we successfully virtualized Philips Hue lamp systems and a virtual face detector by using specific ThingVisors in the current implementation of VirIoT [25]. Demonstrative videos of VirIoT can be found at [32].

### 3.3. Virtual Silos

VirIoT tenants can instantiate vSilos and connect to them any vThing offered by ThingVisors on-demand. A vSilo includes two internal Controllers (IoT and HTTP) and the two corresponding Brokers (Figure 2). The Controllers configure their relative Brokers and the IoT Broker also carries out information model translation for context data.

When a vThing is connected to a vSilo, the IoT Controller begins to receive context data, and it begins translating and inserting such data into the IoT Broker. At the same time, the HTTP Controller configures the access policy of the HTTP Broker so as to unlock forwarding of HTTP requests for vThing’s generic contents into the platform.

The current implementation of VirIoT allows instantiating oneM2M vSilos that internally use a Mobius IoT Broker [33], NGSIv2 vSilos that internally use an Orion IoT Broker [34] and many NGSI-LD vSilos that use different NGSI-LD brokers [35,36]. Each Broker offers both publish-subscribe and request-response APIs so the tenant can use a vSilo to develop both event-driven and synchronous (e.g., on demand data sensing) IoT applications.

### 3.4. MQTT and HTTP Distribution Systems

The internal communications among VirIoT services use a data distribution system that implements topology routing, in-network caching and multicasting to reduce latency and wide area network traffic. Communication concerns both the control and data planes. Control plane communications involve the exchange of control messages used to configure VirIoT services (see documentation in [25]); data plane communications involve the exchange of messages carrying context data and generic HTTP contents of vThings.

VirIoT adopts two mechanisms for the internal communications: publish-subscribe message distribution for control data and context data and HTTP-based request/response mechanism for the retrieval of generic contents.

#### 3.4.1. MQTT Distribution System

Control plane messages and data plane messages carrying the context data of vThings are exchanged via a topic-based publish-subscribe distribution system that uses the MQTT technology [27]. As shown in Table 3, each service is associated with an input (c_in) and an output control topic (c_out). An input control topic is used by a service to receive control messages. An output control topic is used by a service to send control messages to all other interested services. Context data are transferred on input (data_in) and output (data_out) data topics, similarly to the control plane, but for data flows. For instance, the context data pieces produced by a vThing are sent on the data output topic of the vThing. vSilos that contain the vThing are subscribers of this data output topic, therefore, they receive the context data and insert them in their Brokers. A data input topic is, for instance, used by a vThing associated with an actuator to receive actuation-commands sent by vSilos.

Now that we have briefly described how MQTT is used by the services, we present our implementation and the advantages brought up by this implementation. The MQTT distribution system consists of a set of MQTT Brokers that form a single (distributed) MQTT cluster [37]. There is one Broker per VirIoT zone, i.e., per data center or edge node used by the platform (Figure 4). Each VirIoT service (ThingVisor, vSilo, etc.) is connected to the MQTT Broker of its zone, and we exploited the k8s service topology routing feature to properly steer MQTT connections.

When any VirIoT service publishes a message to a topic, the MQTT Broker of the zone forwards a copy to (i) connected services of the zone that are subscribers of the topic and (ii) to remote MQTT Brokers of other zones that have at least one interested subscriber. The remote MQTT Brokers, in turn, forward a copy of the message to all connected subscribers. Overall, the message delivery follows a *topology-based* multicast distribution tree rooted at the publisher; intermediate nodes are the MQTT Brokers; and leafs are the subscribers.

The use of such a MQTT-based distribution system for context data and control messages coupled with our approach of combining MQTT clustering and k8s topology routing offers many advantages:

- Decoupling publishers (e.g., ThingVisors) from subscribers (e.g., vSilos) supports the highly dynamic nature of VirIoT, where vSilos are created/destroyed, vThings added/removed and ThingVisors added/removed, etc.;
- Data latency among ThingVisors and vSilos is very low due to the push-based delivery model and the persistence of MQTT connections;
- Inter-zone traffic is reduced due to topology-based multicasting.

We used the VerneMQ Broker [38] to implement the MQTT cluster, and although the cluster configuration is designed to work with Brokers located in a single data center, VirIoT uses Brokers running in different data centers/zones.

#### 3.4.2. HTTP Distribution System

The HTTP Distribution System allows tenants to access the generic contents offered by vThings. The system consists of a single (distributed) cluster of HTTP proxies, and there is a proxy for each VirIoT zone (Figure 4). When a tenant wants to access a generic content offered by a vThing (e.g., a JPEG image from a virtual camera), she sends an HTTP GET to a vThing-specific endpoint exposed by the HTTP Broker of her vSilo. The HTTP Broker forwards this requests to the HTTP proxy of its zone. In turn, the HTTP proxy relays the request to the vThing. If allowed by the HTTP cache-control headers, the returning content is cached by the zone HTTP proxy to speed up subsequent requests for the same content and to reduce inter-zone traffic. In the case of concurrent requests, only one of them is forwarded by the HTTP proxy to the ThingVisor, thus implementing a multicast distribution for concurrent accesses to the same content, such as a MPEG DASH live streaming session.

We used the NGINX proxy with collapsed forwarding and caching to implement the HTTP Distribution System and (again) the k8s service topology routing feature to route the vSilo HTTP GETs to the HTTP proxy of the zone.

## 4. Performance Evaluation

The main objective of VirIoT is to provide tenants with vSilos, which represent our concept of IoT Infrastructures offered as-a-service while supporting many IoT standards and handling related interoperability issues. These “functional” capabilities are shown by a demonstration video in [28]. In this section, we focus on performance aspects and report a performance evaluation that has two goals: first, to show the efficiency and scalability of VirIoT in providing vThing data to vSilos; second, to assess VirIoT’s ability to integrate different IoT standards into a single platform while also carrying out a fair comparison of current open source implementations of various IoT Brokers.

We have deployed an instance of the VirIoT platform that involves k8s nodes located in two data centers: one in Europe (EU zone) and one in a Japan (JP zone), both belonging to Microsoft Azure Cloud. The nodes of the two data centers are interconnected by a EU-JP virtual private network link, and the supporting virtual machines have (nearly) synchronized clocks. This deployment is that of Figure 4, assuming zone 1 is within the EU data center and zone 2 is within the JP data center.

To measure performance, we used a *producer* that sends context data and generic contents to a set of *consumers*. Producer and consumers are interconnected by VirIoT as follows.

The producer is an application that periodically generates context data items (2 msg/s) containing a sequence number and a timestamp and sends them to a ThingVisor named Relay-ThingVisor by using an HTTP REST API. Furthermore, a HTTP server connected to the Relay-ThingVisor is also part of the producer and offers two generic contents: a 100 MB dummy file and a 2 Mbit/s MPEG DASH encoded video (Big Buck Bunny) having a 4 s segment length. The Relay-ThingVisor implements a simple Virtual Sensor, called Relay-vThing, which relays back into the system context data received from the producer. In addition, the Relay-vThing also provides the HTTP access to generic contents hosted by the producer’s HTTP server.

A consumer is an application that connects to a vSilo, which contains a Relay-vThing. A consumer is able to (i) receive context data and compute the end-to-end transfer delay, (ii) download the 100MB dummy file and (iii) participate in a live streaming session of the MPEG DASH video. We have developed several consumer applications for the different types of IoT Brokers (NGSIv2, NGSI-LD and oneM2M) offered by the vSilos of VirIoT.

### 4.1. Efficiency and Scalability of Data Distribution

**Figure 9:**
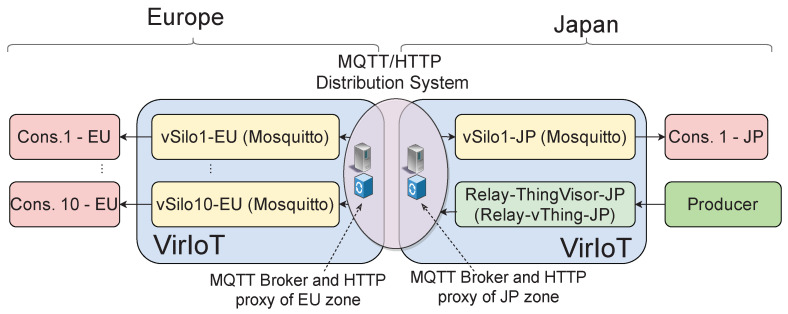
Test scenario 1.

Figure 9 describes the test scenario we used to perform this analysis. We deployed a Relay-ThingVisor-JP in the JP data center and we connected a producer application to it; its vThing is the Relay-vThing-JP. We deployed 11 *Mosquitto* vSilos: 10 vSilos located in the EU data center (vSilo*i*-EU with 1≤i≤10) and one vSilo (vSilo1-JP) in the JP data center. These vSilos contain an IoT Broker which is a simple MQTT Mosquitto server, which transmits the NGSI-LD context data received from the vThings to external MQTT topics that tenant applications can subscribe to. Finally, we have connected to each vSilo’s IoT Broker a consumer application located in the same data center as the vSilo.

We connect Relay-vThing-JP to vSilo1-JP at the beginning of the test. Then, we connect Relay-vThing-JP to EU vSilos (vSilo*i*-EU) in a time sequence to monitor the impact of these configuration changes on the inter-zone (EU-JP link) network traffic of context data. Specifically, Relay-vThing-JP is connected to the *i*th EU vSilo at time 60+20i sec.

**Figure 10:**
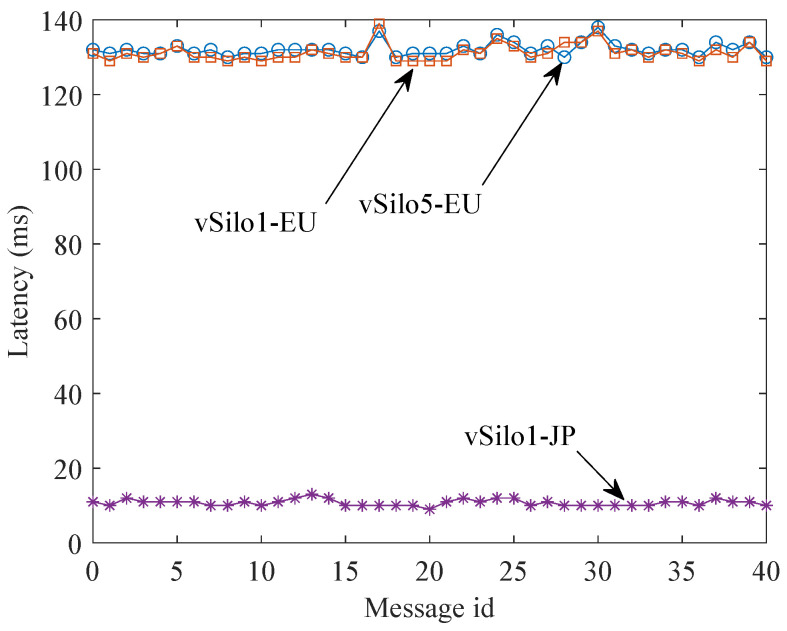
Latency of context data.

Figure 10 shows the end-to-end delay between the production and the consumption of a context data item. To simplify the plot, on the EU side, we have only included the performance measured by Consumer 1 (attached to vSilo1-EU) and Consumer 5 (attached to vSilo5-EU); performance through the other EU vSilos is similar. Data flowing through vSilo1-JP experiences a latency on the order of 10 ms because Relay-vThing-JP is located in the JP data center; therefore, both services are automatically connected to the MQTT Broker of the JP zone (Figure 9) making the latency very low. On the contrary, the EU vSilos experience a data latency on the order of 130 ms because the context data pieces pass through the JP MQTT Broker and then through the EU MQTT Broker before finally reaching vSilos.

**Figure 11:**
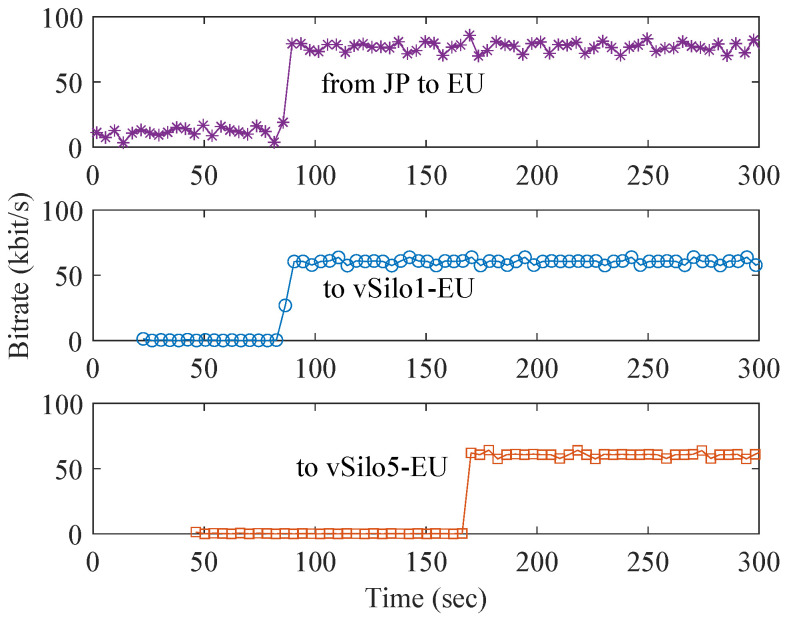
MQTT traffic of context data.

Figure 11 shows the bitrate of different traffic flows measured at the EU MQTT Broker during the test. The JP-to-EU traffic begins to grow when Relay-vThing-JP is connected to the first EU vSilo (at sec. 80), and consequently context data begin to be transferred between the two data centers. After that, this traffic remains constant while the other EU vSilos connect to Relay-vThing-JP and begin receiving its traffic (e.g., vSilo5-EU at sec. 160) because the EU MQTT Broker acts as a multicast split point serving all vSilos located in the EU data center. We notice that the JP-to-EU traffic is higher than that of each vSilo because the MQTT clustering background traffic is about 15 kbit/s (e.g., for monitoring cluster status, etc.), which is observed even before the first EU vSilo is connected to the Relay-vThing-JP at sec. 80.

Overall, these two plots show the effectiveness of the MQTT Distribution System to optimize data latency and network traffic of context data by creating a multicast distribution tree that follows the network topology.

**Figure 12:**
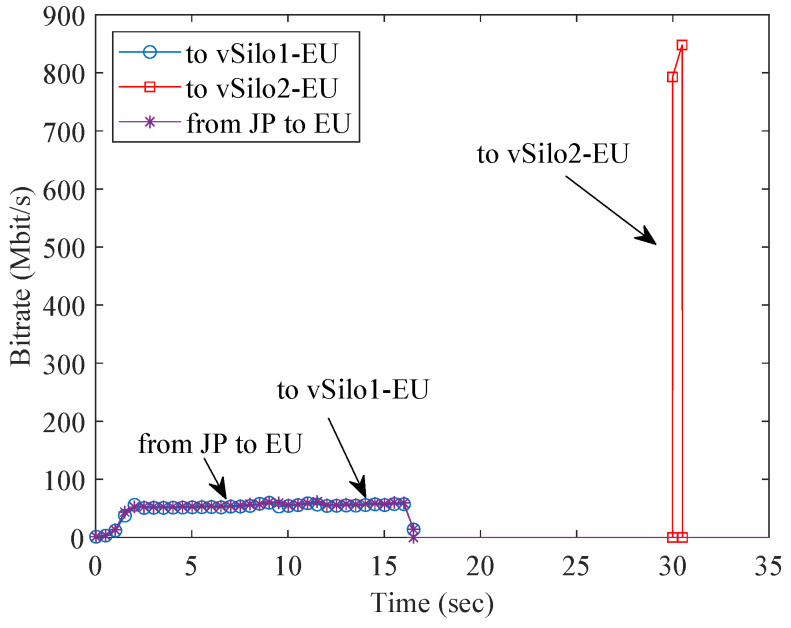
HTTP traffic during the download of a generic content.

Now let us evaluate the performance of the HTTP Distribution System. First, we analyze the caching capability using two EU vSilos. At the beginning of the test, the consumer application connected to vSilo1-EU downloads the dummy file (100 MB) provided by Relay-vThing-JP. Another consumer application, connected to vSilo2-EU, repeats the download after 30 s. Figure 12 shows the bitrate of the different traffic flows measured at the EU HTTP proxy. During the first download, there is traffic flow from JP to EU coming from Relay-vThing-JP to the EU HTTP proxy. From here, this traffic is relayed to vSilos1-EU. The bit rate is limited by the bandwidth of the JP-EU link: it is in the order of 54 Mbit/s, and the download takes about 13 s. The dummy file is cached by the EU HTTP proxy; hence, during the second download, there is no traffic from JP but only from the cache of the EU HTTP proxy to vSilo2-EU. They are in the same data center, so the bit rate is very high, and the download takes only 1 s.

**Figure 13:**
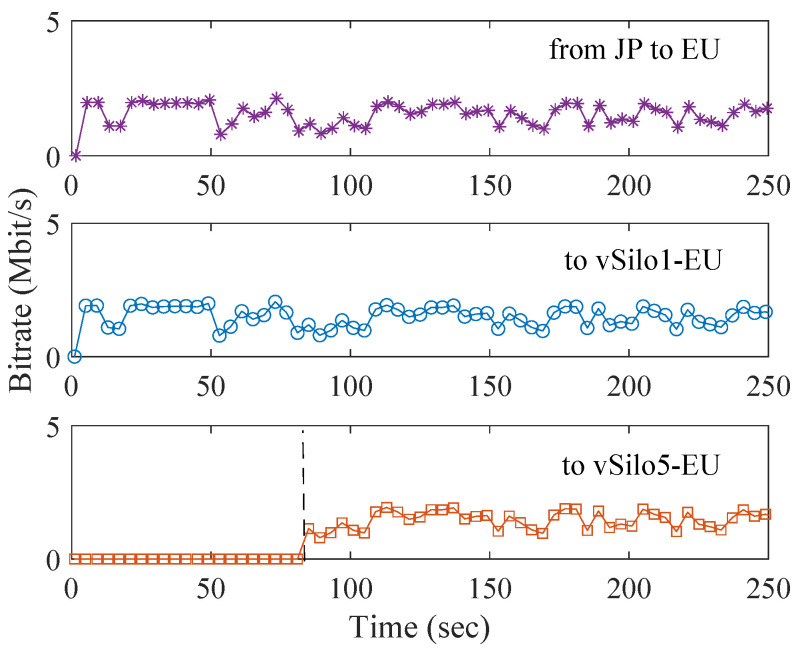
HTTP traffic during a live streaming session.

The second assessment we make on the HTTP Distribution System is its ability to efficiently distribute live contents. We used five EU vSilos. Each one of them is used by a consumer application (mimicking a video player) which joins an MPEG DASH live video streaming session provided by Relay-vThing-JP. Consumer *i* connected to the *i*th vSilo joins at time 16∗(i−1) seconds. Figure 13 shows the bitrate of the different traffic flows measured at the EU HTTP proxy. Traffic starts flowing from JP when, at sec. 0, the consumer of the first EU vSilo joins the live streaming session. As expected, when the consumers attached to the other vSilos (e.g., vSilo5-EU) start watching the video, traffic from Japan does not change because the EU HTTP proxy is acting as a multicast split point for the vSilos located in the EU data center.

Overall, Figure 12 and Figure 13 show the effectiveness of the HTTP Distribution System at optimizing network traffic of generic contents by exploiting caching and by creating a multicast distribution tree that follows network topology.

### 4.2. Comparison of Open-Source IoT Brokers

We used the VirIoT platform to compare, side-by-side, the performance of several open-source IoT Brokers by running them within specific vSilos. The IoT Brokers we considered are as follows: Mobius (v2) oneM2M [33], NGSIv2 Orion [34], NGSI-LD OrionLD [35] and NGSI-LD Scorpio [36]. We also included a Mosquitto vSilo in the test. Each vSilo is connected to a Relay-vThing-EU implemented by a Relay-ThingVisor-EU and deployed in the EU data center, along with its producer application.

To measure data latency, we connected a different consumer application to each vSilo’s IoT Broker. These applications receive context data from Relay-vThing-EU by using the publish/subscribe services offered by the vSilo’s IoT Brokers, thus avoiding any polling operation and minimizing the transfer delay. NGSIv2 and NGSI-LD publish/subscribe services are via HTTP; the consumer tells the IoT Broker the notification URI where it wants to receive data. In addition to HTTP, oneM2M technology also supports MQTT binding for publish/subscribe services: the consumer subscribes to a topic where the oneM2M Broker (Mobius) publishes context data.

**Figure 14:**
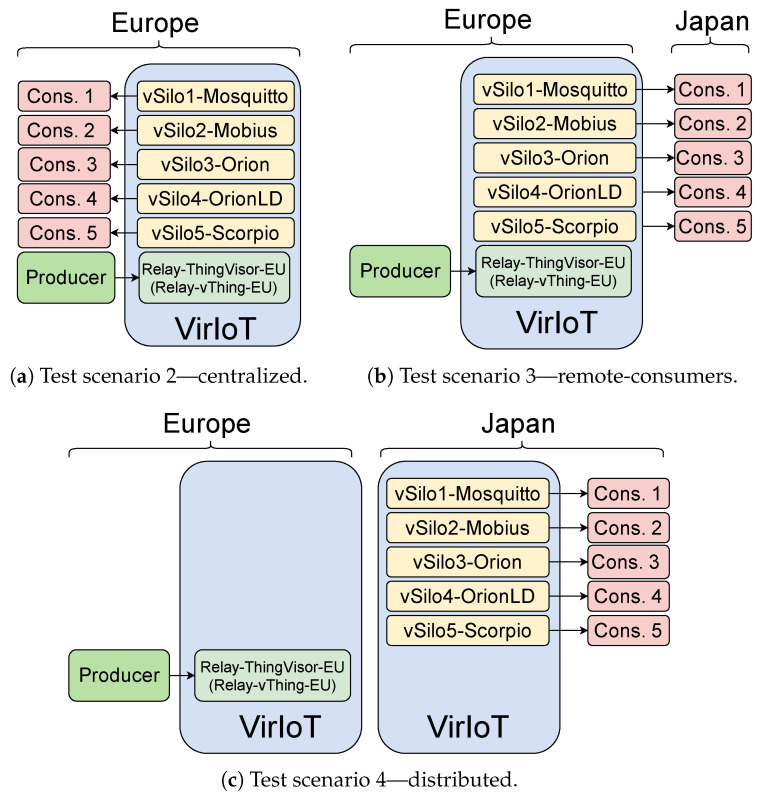
Test scenarios used in the comparison of IoT Brokers.

We analyze three scenarios, as depicted in Figure 14. Figure 14a represents a centralized deployment of VirIoT where the entire platform is installed in a single EU data center. Producer and consumers are located in the same data center too. In Figure 14b, we have the same EU centralized deployment, but we have moved the consumers away from their vSilos from Europe to Japan. Finally, in Figure 14c, we leave the consumers in Japan, but we consider a distributed VirIoT deployment that also includes a JP data center, and we move the vSilos there so that they can be closer to the consumers.

**Figure 15:**
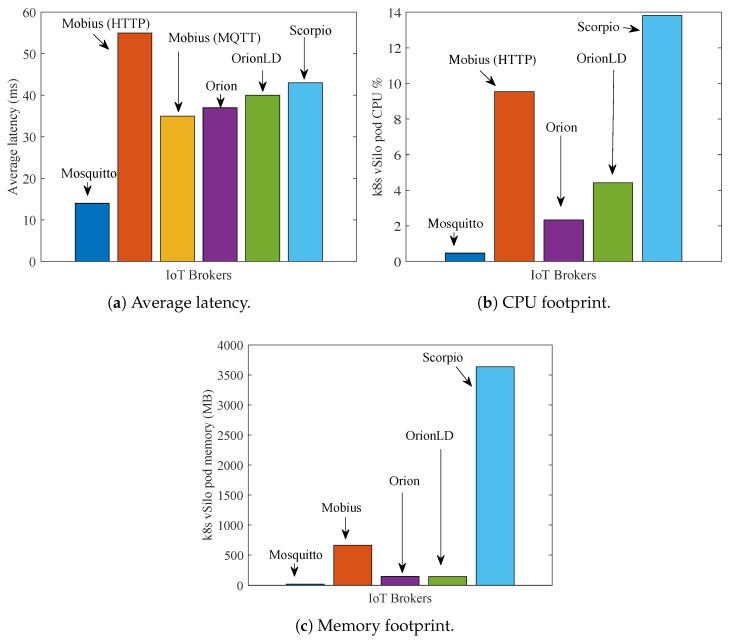
Performance of vSilos with different IoT Brokers for test scenario 2 (centralized).

Let us start discussing the results we have obtained in the centralized scenario of Figure 14a. Figure 15a shows the related average latency of context data for different IoT Brokers. For the oneM2M Mobius Broker, we considered both transport options: HTTP and MQTT. As expected, Mosquitto-based vSilo is the fastest. However, this is not a fair comparison because this vSilo does not really offer tenants the services of an IoT integration platform as the other vSilos do; in fact, the Mosquitto vSilo simply forwards NGSI-LD data received from vThings, onto external topics. For this reason, the difference in delay performance between Mosquitto and the other vSilos can be considered as a measure of the processing effort required to virtualize a fully featured integration platform rather than a simple pass-through service.

Performance comparison of the other vSilos reveals that the oneM2M Mobius Broker is a bit slower than the others with HTTP transport, but its latency is reduced when using MQTT. This HTTP/MQTT difference is motivated by the fact that with HTTP it is necessary to open and close a TCP connection for each data piece from vSilo’s IoT Broker and the consumer application; indeed, HTTP persistent connections do not apply for publish/subscribe callbacks; all of the Brokers close the connection after data delivery. With MQTT, the TCP connection is persistent; thus, there is no RTT penalty. NGSIv2 Orion and NGSI-LD OrionLD have very similar latency. NGSI-LD Scorpio is a little bit slower. In general, all vSilos have latency in the range of 35–55 ms.

Figure 15b,c show the CPU and memory footprint of vSilos during the test. For all Brokers, CPU usage is quite limited. Of the NGSI-LD vSilos, Scorpios have a much larger memory footprint than OrionLD. This is motivated by the fact that Scorpio is based on Java, is designed for systems with many users and therefore uses many components (Apache Kafka, PostgreSQL, etc.) that are memory hungry. Conversely, OrionLD is lighter, uses C++ and MongoDB.

**Figure 16:**
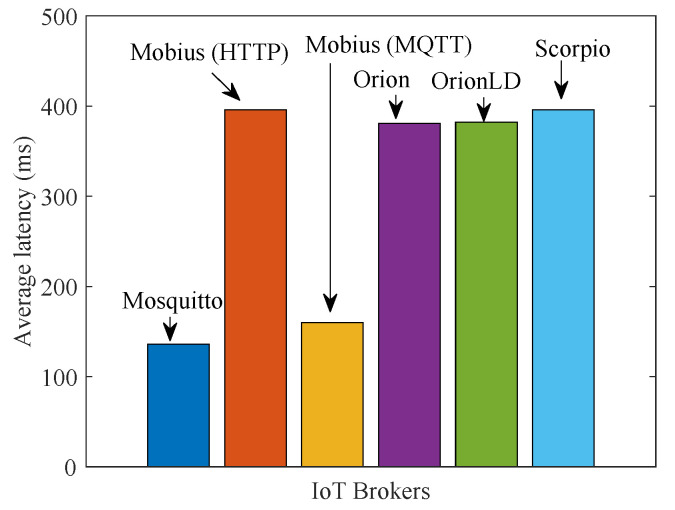
Average latency for test scenario 3 (remote-consumers).

We now report on the latency figures we obtained in the two remaining scenarios: remote-consumers and distributed. Figure 16 refers to the remote-consumers scenario of Figure 14b and shows that, when consumers are moved away from their IoT Brokers, Brokers using an HTTP API (Mobius with HTTP, Orion, OrionLD and Scorpio) suffer a significant latency increase due to the need to establish a TCP connection for each data transfer. Brokers using MQTT (Mosquitto and Mobius with MQTT) do not have this problem as they use persistent TCP connections where data are immediately pushed.

**Figure 17:**
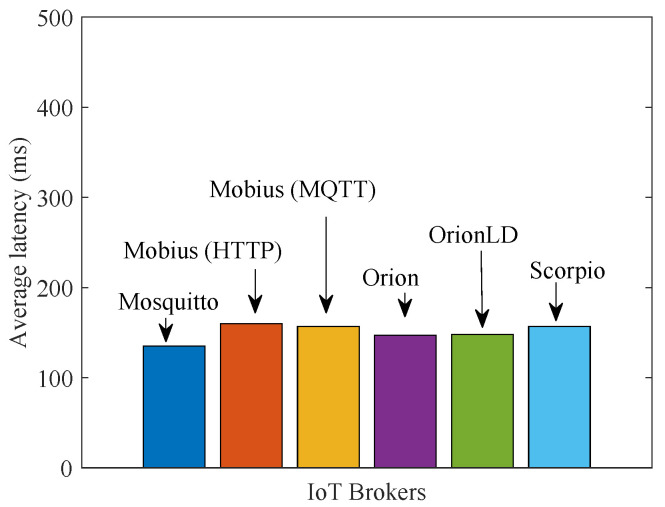
Average latency for test scenario 4 (distributed).

Figure 17 refers to the distributed-platform scenario of Figure 14c, and the comparison with Figure 16 shows that the delay penalty of IoT Brokers using HTTP APIs can be reduced by leveraging VirIoT ability to allocate services close to producers and consumers, specifically vSilos in Japan and ThingVisor in Europe. This shortens the distance between the involved HTTP endpoints (Producer–ThingVisor; IoT Broker–Consumer) and makes end-to-end data transfer faster.

Finally, we analyze a typical drawback of virtualization technologies, which is the performance penalty of a virtualized system compared to a native, i.e., non-virtualized system. To this end, we carried out latency measurements of native systems, each composed of an IoT Broker (Mobius, Orion, etc.) and a consumer/producer pair directly connected to it. All three components, Producer-Broker-Consumer, are located in the same EU data center. The equivalent but virtualized configuration to be used for a performance comparison is the one shown in Figure 14a.

**Figure 18:**
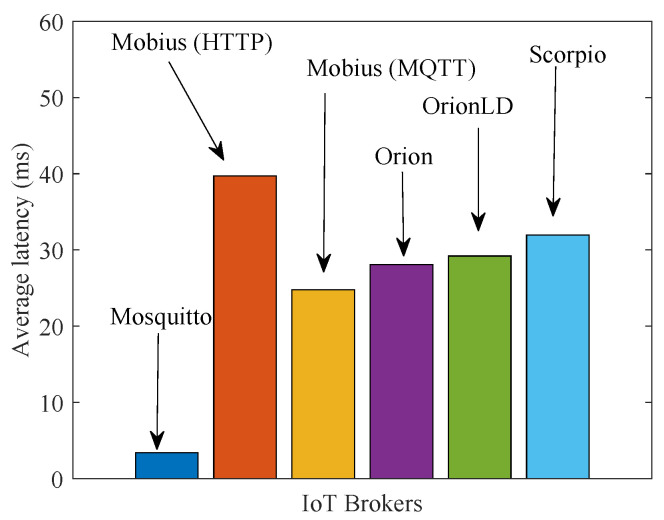
Average latency of native systems (to be compared with Figure 15a).

Figure 18 shows the data latency achieved for native systems. By comparing these results with those in Figure 15a, we observe a delay penalty caused by virtualization on the order of 10 to 15 ms. This is due to the fact that, in VirIoT, a context data piece goes through a ThingVisor, the MQTT Distribution System and a vSilo IoT Controller, eventually arriving at the vSilo IoT Broker (Figure 2). In a native system, the producer directly injects context data into the IoT Broker. These additional virtualization steps increase data latency because they introduce processing delays that, however, are implementation-dependent. The implementation is currently in a preliminary stage and uses interpreted programming languages (Python and JavaScript) rather than faster compiled languages; therefore, we argue that this delay penalty can be reduced.

Overall, the entire performance evaluation showed that VirIoT is a Cloud of Things platform that works efficiently and is able to leverage infrastructures made up of many cloud/edge sites and sensors/actuators while limiting site-to-site and thing-to-site bandwidth consumption and data latency and offering very low virtualization overhead compared to native systems. The platform is able to integrate different standard IoT Brokers, and their comparison within VirIoT showed that, when clients are far away, Brokers supporting MQTT access provide rather low latencies compared to those offering HTTP access. In terms of latency, memory and CPU footprint, brokers from the Orion family performed slightly better when clients were close to their vSilos. For distant clients, Mobius with MQTT has lower latency because the other Brokers only offer HTTP access.

## 5. Conclusions

We propose a new form of cloud services for IoT that mimics the infrastructure as a service but addresses Internet of Things instead of computing. Just as cloud computing offers virtual machines made of virtual hardware and an operating system, our Cloud of Things, VirIoT, offers Virtual Silos made of Virtual Things and standard IoT Brokers.

An initial challenge we found when designing the platform concerned *interoperability* at the API, data model and device levels. Our position was not to introduce yet another data model and/or API that users must comply with, but instead we wanted to create a system that can integrate many IoT technologies in an evolutionary manner. Different sensors/actuators can be connected to the platform by inserting the ThingVisors that interact with them using their APIs and translating their data into VirIoT’s internal NGSI-LD format. This ingress translation, however, does not force the consumers of this data to use NGSI-LD for their applications. In fact, different IoT Brokers technologies (oneM2M, NGSI-LD, NGSI, etc.) with their data models and APIs can be added to the system and offered to users inside their vSilos, which internally implement the conversion rules from NGSI-LD. This *double* translation of data models and API (from real device to NGSI-LD to IoT Broker) has allowed VirIoT to integrate heterogeneous devices and IoT Brokers. Moreover, updating/upgrading the system with new technologies, e.g., new ThingVisors or vSilos types, can be performed continuously and without changing other running services because VirIoT is a microservice architecture.

The *scalability* of the system with respect to the number of users/devices has also been a challenge. With reference to a native, non-virtualized system made by a Broker, we note that the latter can support more or fewer users depending on its implementation. However, it is a single entity; thus, it is more prone to suffer from scalability issues. Similarly, a proxy/hub agent that connects many IoT devices to cloud services may have the same problem; likewise, a IoT device used by many clients (e.g., via HTTP) may have issues due to possible bandwidth or processing limitations. VirIoT addresses these user/device scalability issues by using different IoT Brokers (within vSilos) per tenant and different ThingVisors per IoT device. These components can be run in parallel on a cluster of cloud computing resources, thus allowing horizontal scalability of VirIoT services regardless of the specific IoT Broker or device technology. Moreover, IoT devices connected to VirIoT have only one client, which is their ThingVisor, thus limiting their bandwidth and processing requirements. In fact, VirIoT takes care of distributing the device’s data to many users and handling any API/data model translation. Furthermore, the data distribution system has also been made horizontally scalable and bandwidth-saving by using a distributed system of MQTT brokers and HTTP caches that implements topology-aware service routing. The system database also supports horizontal scalability by being NoSQL based, and the Master Controller can be replicated to support a possible increase in load. Overall, VirIoT is a horizontally scalable architecture that also limits bandwidth consumption for data transmission and does not require onerous computation requirements to the real devices connected to it.

A final challenge was the management of virtual actuators. Indeed, while the Sensing-as-a-Service concept has been previously addressed in the literature and in projects, the virtualization of actuators considering data interoperability issues and the need to support heterogeneous use cases has been a new innovation challenge that we solved by proposing a command-oriented actuator control strategy and a new concept of actuation QoS.

Future work may include deeper security and threat analysis; possible exploitation of semantic annotations (already carried by internal NGSI-LD data) for semantically oriented control operations, such as adding a vThing to a vSilo only by describing the semantic properties of the vThing or data (e.g., “temperature of Rome”) of interest rather than its vThingID; and, finally, even though we deployed the platform by using many clouds/edge data centers connected by VPN links, we noticed that when the cloud infrastructure is dynamic, e.g., IP addresses change, the Kubernetes configuration we used has problems. Thus, supporting VirIoT services with a dynamic underlying infrastructure is a challenge for future investigations.

Inspired by the NIST definition of Cloud Computing [24], we conclude this paper by summarizing the essential characteristics our Cloud of Things has:

- *On-demand self-service*: A consumer can unilaterally provision IoT resources, such as Virtual Things, IoT Broker and Virtual Silos;
- *Broad IoT access*: IoT resources are available over the network and accessed through a plethora of heterogeneous IoT standard technologies, such as those specified by NGSI, NGSI-LD, oneM2M, etc;
- *Broad support of IoT devices*: We support heterogeneous IoT sensors and actuators producing or consuming context data and generic, large-size HTTP contents;
- *Resource pooling*: Underlying computing and IoT resources (real things, open data, etc.) are pooled to serve multiple consumers by means of different kinds of virtual resources that are dynamically assigned and reassigned according to current demand from consumers.

## Figures and Tables

**Table 1:** Summary of related works.

Ref.	Platform	Main Idea	Comparison
[2]	IoT-IaaS	Sensor sharing	No actuation; IoT data moved to centralized database
[16]	Stack4Things	Sensing and actuation as a service	No conversion to “neutral format”; hence, no cross-domain interoperability
[3]	AWS Greengrass	Tight integration of IoT devices with the cloud	Does not provide IoT infrastructure itself or permit sharing of IoT infrastructure by multiple tenants
[18]	Virtual Sensors	Abstracting data from physical sensors	No containerized approach; no actuation
[19]	An event-driven sensor virtualization approach	Information obtained from IoT devices is enriched using semantic representations	No standards-based data model is used; custom concepts were defined
[20]	Cloud of Things	Use of a middleware layer for storing and processing IoT data	No actuation; no virtualization of services; no conversion to “neutral format”; hence, no cross-domain interoperability
[21]	Sensor Cloud	Sensors spread in a huge geographical area can connect together and be employed simultaneously by multiple users in a many-to-many configuration	No actuation; focus on integrating distributed Wireless Sensor Networks of homogeneous type
[22]	Enhancing Dependability of Cloud-based IoT Services through Virtualization	Enhancing two desired service dependability features, reliability and availability, at the application layer	Narrow focus on dependability
[11]	iTaaS	Solution based on micro-services to support data collection from IoT devices to a gateway on a real time basis and data sharing, storage and processing	No actuation; no support for bulk HTTP data streams
[15]	Smart Virtualization for IoT	Builds distributed virtual systems that include the benefits of the cloud, fog and dew computing to provide services directly at the edge level	No conversion to “neutral format”; hence, no cross-domain interoperability
[12]	Virtual Fog	A layered framework grounded on object virtualization, network function virtualization and service virtualization	No conversion to “neutral format”; hence, no cross-domain interoperability

**Table 2:** NGSI-LD to NGSIv2 and oneM2M. For oneM2M, the Application Entity (AE) name is equal to the vThing name.

NGSI-LD	NGSIv2	oneM2M
Entity	Entity	Top-level Container (cnt)
Property/Relationship	Attribute	Sub Container
Entity ID	Entity ID	Top-level Container resourceName (rn)
Entity Type	Entity Type	Top-level Container Labels (lbl)
Property/Relationship Name	Attribute Name	Sub Container resourceName
Property/Relationship Block	Attribute block	Content Instance (cin) of the sub Container

**Table 3:** System Topics.

Topic	Description
vThing/<vThingID>/ {c_in,c_out,data_in,data_out}	Used by a ThingVisor to send (c_out) and receive (c_in) control messages related to the vThing (e.g., removal, change of configuration parameters, etc.) or to publish context data items of a vThing data_out or to receives actuation command data_in.
TV/<TVID>/{c_in,c_out}	Used by a ThingVisor to send (c_out) or receive (c_in) control messages related to the whole ThingVisor (e.g., pause, remove, activate vThing, etc.).
vSilo/<vSiloID>/{c_in,c_out,data_in}	Used by the vSilo controller to send (c_out) or receive (c_in) control messages related to the specific Virtual Silo (e.g., add vThing, remove vThing, etc.) or to receive (data_in) Virtual Silo specific data (e.g., actuation feedback, initial context synchronization, etc.).
master/{c_in,c_out}	Used by the master-control to send (c_out) or receive (c_in) control messages related to the system configuration.
